# Comparative effect of thymus vulgaris and ibuprofen on primary dysmenorrhea: A triple-blind clinical study

**Published:** 2014

**Authors:** Hajar Salmalian, Roshanak Saghebi, Ali Akbar Moghadamnia, Ali Bijani, Mahbobeh Faramarzi, Fatemeh Nasiri Amiri, Fatemeh Bakouei, Fereshte Behmanesh, Reza Bekhradi

**Affiliations:** 1 Fatemeh Zahra Infertility and Reproductive Health Research Center, Department of Midwifery, Babol University of Medical Sciences, Babol, Iran.; 2 Faculty of Traditional Medicine, Babol University of Medical Sciences, Babol, Iran.; 3 Department of Pharmacology, Babol University of Medical Sciences, Babol, Iran.; 4 Social Determinants of Health Research Center, Babol University of Medical Sciences, Babol, Iran.; 5 Department of Midwifery, Babol University of Medical Sciences, Babol, Iran.; 6 Research Development Unit of Barij Essence Kashan, Iran.

**Keywords:** Thymus vulgaris, Primary dysmenorrhea, Iboprofen

## Abstract

***Background: ***Dysmenorrhea is one of the most common medical problems in gynecology causing several problems in the personal and social life of women. This study was conducted to compare the effect of thymus vulgaris and ibuprofen on the treatment of primary dysmenorrhea

***Methods:*** This clinical study was conducted on 84 students of Babol University of Medical Sciences with primary dysmenorrhea. The students were randomly assigned to three groups receiving thymus vulgaris, ibuprofen and placebo. In all three groups, with the beginning of pain, 200 mg capsules and 25 drops of essential oil were given every 6 hours for two consecutive cycles. Pain intensity used the visual scale before and one hour after each dose for 48 hour after starting medication. The data were collected and analyzed. This study was registered in the Iranian Registry of Clinical Trial (www.irct.ir) with registration number ID: IRCT201101245683N1

***Results:*** The mean age of participants was 20.5±1.8 years. Both thymus vulgaris and ibuprofen were effective to reduce the pain severity of dysmenorrhea. Before treatment, the mean pain intensity in thymus vulgaris, ibuprofen and placebo groups were 6.57±2.02, 5.30±2.23 and 6.18±1.78, respectively and after treatment decreased to 1.21±1.06, 1.48±1.62 and 3.54±2.26, respectively. Reduction of pain severity was not statistically significant between the two medications, however it was significant for each drug compared with placebo (p<0.001).

***Conclusion: ***The results suggest that thymus vulgaris as well as ibuprofen can be effective in reducing the severity of pain and spasm in primary dysmenorrhea.

Primary dysmenorrhea is the most common complaint in a large number of women who experience this condition (1). The pain usually begins a few hours before bleeding and lasts 32 to 48 hours (2, 3). According to the report of WHO, the prevalence of the disease is 1.7-97% (4). In a study in Iran, the prevalence of primary dysmenorrhea was 71% as 15% of students were absent 1 to 7 days during the school year due to dysmenorrhea (5). Furthermore, dysmenorrhea is the most common cause of absence from school and work in women, causing 600 million work hours and 2 $ billion loss annually in the US (6-8). Although the certain cause of primary dysmenorrhea is unknown, one regarded notion is overproduction of endometrial prostaglandins, therefore, reducing the production of prostaglandin treatments are thought to be effective (9). 

The most common dysmenorrhea treatment is non-steroidal medications which come with side effects such as headache, dizziness, dysuria, sleepiness, loss of appetite, nausea, acne, increased acute asthma, vomiting and gastrointestinal bleeding (10). Thymus vulgaris is one of the herbs that is used in the treatment of dysmenorrhea. It is a plant of the Labiatae family with the scientific name "Zataria multiflora". The active material of thymus vulgaris oil is thymol and carvacrol. In traditional medicine; various attributes have been mentioned for this herb; such as assisting digestion as thymus vulgaris syrup helps in indigestion. Its antispasmodic effect on smooth muscles and also anti-microbial properties are the most common effects of this herb (3). 

Unlike NSAIDs medications, thymus vulgaris does not cause gastrointestinal complication and also relieves gastrointestinal disorders such as ulcers, indigestion, constipation, flatulence, and asthma (11). As the cost of treatments is significant for the patients and government and also some of them are partial and unsatisfactory, people increasingly turn to the methods of complementary and alternative medicine (12). Although, there have been various methods recommended to cure dysmenorrhea, but the side effects of these medications and the high cost of importing their raw materials encouraged us to investigate and compare the positive effect of thymus vulgaris and ibuprofen on dysmenorrhea.

## Methods

This randomized clinical study was conducted in Babol University of Medical Sciences. To investigate the research hypothesis, the investigators performed a triple-blind clinical study based on the consent statement and Declaration of Helsinki. The study population consisted of 84 students (age 18-24) of Babol University of Medical Sciences suffering from primary dysmenorrhea. All the students were single. Primary dysmenorrhea was defined as dysmenorrhea which begins in 1 to 2 years after menarche and pain coincides with the onset of menstruation with no pelvic pain during the rest of the cycle. 

The women with primary dysmenorrhea grade 1 and grade 2 in their current cycle and at least in their past two cycles that did not use no only analgesic during 48 hours before the onset of the research entered the study. Exclusion criteria included the history of abdominal and pelvic surgery, diagnosed liver or kidney disease, having severe stress (such as the loss of a close relative, serious family dispute and experiencing drastic stress during 6 months before the study), doing heavy exercises, history of allergy to thymus vulgaris and ibuprofen, lack of proper use of medicine, failure to record pain intensity and dissatisfaction of treatment.

The sample size for each group was estimated 28 persons based on 1.5 units difference in reduction of pain with thymus vulgaris versus ibuprofen with α and β errors of 0.5% and 0.20, respectively.

After getting the approval of the Ethics Committee of Babol University of Medical Sciences and informing the candidates of the research process and the effects of the selected medicines, the subjects were recruited. They signed the written consent forms to start the study. The information was collected using a questionnaire consisting of four sections: demographic and menstruation history, menstrual hemorrhage checklist, the multidimensional scale - verbal scoring (to determine the degree of dysmenorrhea) and the visual analog scale (to determine the severity of pain). Pharmaceutical Barij essence Company in Kashan prepared all the drops of thymus vulgaris essential oil 2% and placebo drop which our patients used. In order to have the same form of applied medicine in all three groups, they were all given capsules every 6 hours and 25 drops of essence oil.

- The first group: 200 mg ibuprofen (capsule form) + 25 drops of placebo essential oil
- The second group: 25 drops of thymus vulgaris essential oil 2% + placebo capsule
- The third group: 25 drops of essential oil placebo + placebo capsule

All packages of medication were coded by the pharmacists and given to the subjects in three groups; A, B, C. They were asked to take medication on the first day of their menstrual cycle and beginning of pain and continue every 6 hours. They were to record their pain intensity before the start of medication, one hour after each dose of medicine, 24 hours and 48 hours after the beginning of medication on the forms which they were provided. To assess the score of dysmenorrhea and systemic symptoms associated with dysmenorrhea, the used multidimensional measure speech systems in which its scientific validity has been confirmed. The system consists of four scores:

Zero: The absence of dysmenorrhea, so there is no interference with daily activities.

Grade I: Mild menstrual pain that rarely interferes with daily activities, and mild systemic symptoms and needing to analgesia is very low.

Grade II: Moderate pain and daily activities may be disrupted, but there is no need to miss school or work.

Grade III: Severe pain so the person is not able to perform daily activities and severe systemic symptoms are observed (13). 

Measuring pain intensity was performed using the standard visual analogue scale (VAS). The patients were taught to record their pain intensity on a 10 cm line compared to the most intensive pain that they had experienced and continue the treatment for two consecutive cycles. The severity of dysmenorrhea was measured by visual analogue scale (on the 10 cm tape). On this scale, numbers 1-3 represent mild pain, 4-7 moderate pain and 8-10 severe pain. The validity of Multidimensional Scale - verbal and visual pain scale has been confirmed (13).

To assess bleeding, a type of picture chart; pictorial blood loss assessment Chart (PBAC) was used. The horizontal row of chart shows the numbers of days of menstruation and the vertical row reveals blood pads used during menstruation which indicate mild, moderate, and severe. For its calculation, mild bleeding: ratio one, moderate: ratio five and for a pad full of blood: ratio twenty has been considered. For every pad change, the girls marked the same day the PBAC diagram according to the pad glut of menstrual blood. Then, we multiplied the numbers in the corresponding ratio and added the obtained numbers together and the total score was calculated. In this chart, less than 50 was considered mild hemorrhage, ≥50≤80 moderate and more than 80 severe hemorrhage (14). This study was registered in the Iranian Registry of Clinical Trial (www.irct.ir) with registration number ID: IRCT201101245683N1. 

The data were analyzed using SPSS Version 18. Categorical variables were compared by x^2 ^test. For ordinal variables Wilcoxon and Kruskal-Wallis tests were used between the groups. ANOVA repeated measures and Tukey post hoc tests were used for comparing the trend of pain between groups. P<0.05 was considered significant.

## Results

**Figure 1:**
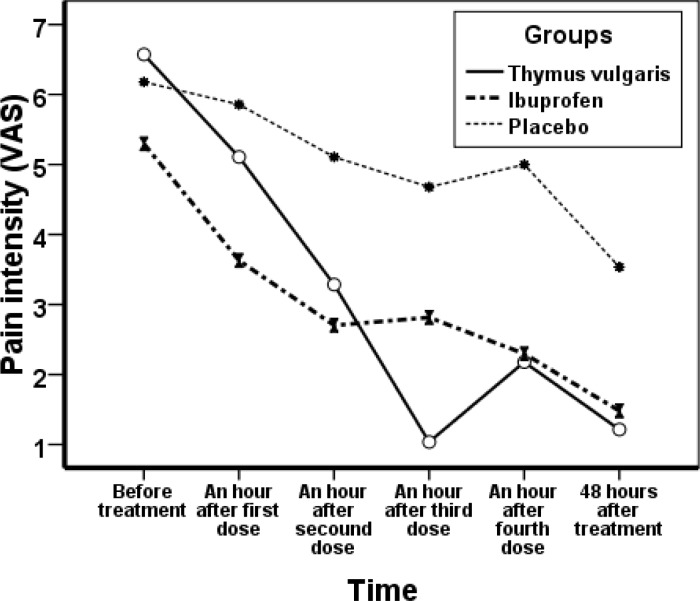
Mean changes in severity of dysmenorrhea groups receiving Thymus vulgaris, Ibuprofen and placebo before and after treatment in the first cycle

**Figure 2:**
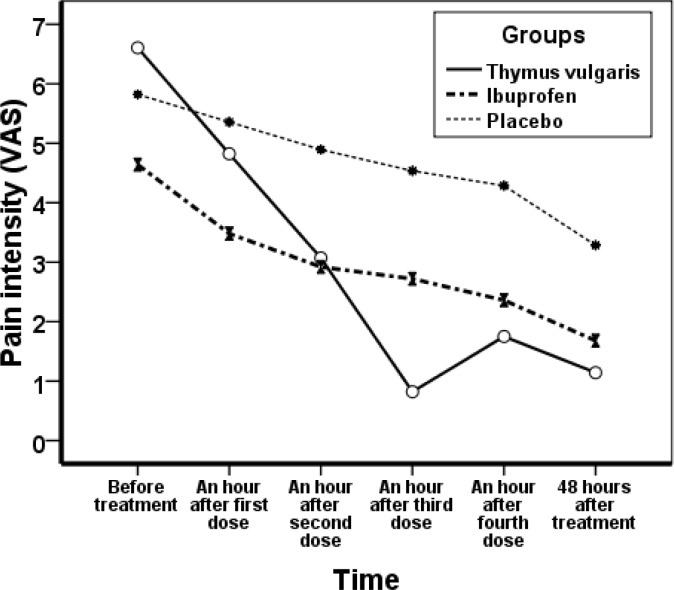
Mean changes in severity of dysmenorrhea groups receiving *T*hymus vulgaris, Ibuprofen and placebo before and after the second cycle.

The mean age of the students was 20.52±1.84 years; the mean age of first menstruation (menarche) was 12. 96±1.34 years and the mean age of start of painful menstruation (dysmenorrhea) was 14.95±1.62 years. All subjects were matched for age, menarche, dysmenorrheal age and body mass index. The mean interval between the two periods (interval cycles) was 28.1±2.79, mean of menstrual duration (bleeding duration) 6.62±1.34 days and their mean of body mass index (BMI) 22.01±3.36. In the thymus vulgaris consumer group, pain score decreased from 6.57±2.02 before treatment to 1.21± 1.06 in the first cycle, and 1.14±1.25 in the second cycle after treatment. In ibuprofen group, pain score before treatment was 5.30±2.23 which changed to 1.48±1.62 in the first cycle and 1.68±2.13 in the second cycle. In placebo group, pain before the treatment was 6.18±1.78 and decreased to 3.45±2.26 in the first cycle and 3.29±2.22 in the second cycle (figures 1 and 2).

Both drugs (thymus vulgaris and ibuprofen) were effective in reducing the severity of dysmenorrheal pain. But the pain decreased more in thymus vulgaris group versus ibuprofen. Pain reduction between these two groups was not significant, but the differences between the placebo group and each of the other groups were statistically significant (table 1). The comparison of bleeding in the three treatment groups before and after treatment showed no significant difference (table 2). 

The severity of clinical symptoms in each of the three treatment groups before treatment and 48 hours after treatment like nausea and vomiting revealed thymus vulgaris and the ibuprofen group had a statistically significant difference (p<0.04, p<0.01, respectively). Also, the variables of diarrhea in ibuprofen group, faint in the thymus vulgaris group and fatigue in the placebo group demonstrated significant differences before and after treatment (table 3).

Comparing the satisfaction of pain relief and symptom reduction after the first and second cycles showed that majority of the subjects in thymus vulgaris group found the results great, ibuprofen group, moderate and the placebo group ineffective (table 4).

**Table 1:** Assessment of the severity of dysmenorrhea in the three groups after treatment

**Criteria**	**Groups**		**Mean Difference**	**p- value** †
Pain intensity in cycle 1	Thymus vulgaris	Placebo	-1.83	0.001
		Ibuprofen	0.2	0.063
Pain intensity in cycle 2	Thymus vulgaris	Placebo	-1.66	0.001
		Ibuprofen	0.07	0.89

† The data were assessed using Tukey’s test.

**Table 2:** Menstruation hemorrhage before and after treatment in the three groups

**Groups**	**Thymus vulgaris**		**Ibuprofen**		**Placebo**	
**Amount of hemorrhage**	**Before %(n)**	**After %(n)**	**Before %(n)**	**After %(n)**	**Before %(n)**	**After%(n)**
Mild	25 (89.3)	27 (96.4)	25 (89.3)	25 (89.3)	27 (96.4)	23 (82.1)
Medium	3 (10.7)	1 (3.6)	2 (7.1)	3 (10.7)	1 (3.6)	4 (14.3)
Sever	0 (0)	0 (0)	1 (3.6)	0 (0)	0 (0)	1 (3.6)
Total	28 (100)	28 (100)	28 (100)	28 (100)	28 (100)	28 (100)
p-value†						

† The data of before and after were assessed using Chi-square test.

NS ; None Significant

**Table 3:** Comparison of clinical symptoms before and 48 hours after treatment in the three groups

**Groups**	**Thymus vulgaris**			**Ibuprofen**			**Placebo**		
**Clinical** **Symptom**	**Before ** **(Mean±SD)**	**48 hr After ** **(Mean±SD)**	**p-value†**	**Before ** **(Mean±SD)**	**48 hr After ** **(Mean±SD)**	**p-value†**	**Before (Mean±SD)**	**48 hr After ** **(Mean±SD)**	**p-value** †
Lower abdominal pain	2.22±0.8	2.14±0.89	NS	2.04 ±0.63	1.89±0.73	NS	2.22± 0.64	2±0.72	0.03
Fatigue	1.78±1.89	1.75 ±0.88	NS	1.54 ±0.74	1.39±0.73	NS	1.69±0.78	1.68±0.98	NS
Nausea and Vomiting	0.62±0.69	0.87 ±0.75`	0.046	0.82 ±0.86	0.61±0.91	0.01	0.71±0.65	0.51±0.57	NS
Lethargy	1.19±0.68	1.32±0.9	NS	1.64±0.62	1.61±0.68	NS	1.63±0.88	1.57±0.79	NS
Diarrhea	0.22±0.5	0.36±0.62	NS	0.57±0.87	0.32±0.67	0.008	0.37±0.62	0.37±0.56	NS
Headache	0.92±0.9	0.96±0.88	NS	0.81±0.78	0.85±0.81	NS	1.12±1.14	1.04±1.01	NS
mood variability	1.63±0.88	1.39±0.73	NS	1.11±0.73	1.18±0.72	NS	1.39±0.99	1.22±0.97	NS
Faint	0.96±0.75	0.64±0.67	0.007	0.36±0.79	0.32±0.69	NS	0.65±0.84	0.54±0.74	NS

† The data of before and after were assessed using Wilcoxon test.

**Table 4:** Rate of satisfaction from pain relief in the three treatment groups

**Groups** **Satisfaction rate **	**After the first cycle of treatment** **%(n)**			**After the second cycle of treatment %(n)**		
	**Thymus vulgaris**	**Ibuprofen**	**Placebo**	**Thymus vulgaris**	**Ibuprofen**	**Placebo**
No effect	0 (0)	4 (1)	57.1 (16)	0 (0)	4.2	57.19 (16)
Slight	0 (0)	28 (7)	17.9 (5)	0 (0)	33.3 (8)	25 (7)
Moderate	28.6 (8)	40 (10)	10.7 (3)	18.5 (5)	37.5 (9)	17.5 (5)
Excellent	71.4 (20)	28 (7)	14.3 (4)	81.5 (22)	25 (6)	0 (0)
Total	100 (28)	25 (100)	100 (28)	100 (27)	100 (24)	100 (28)
p-value†	0.001			0.001		

† The data were assessed using Kruskal-wallis test.

## Discussion

 Excessive production of endometrial prostaglandins is considered as an acceptable theory about primary dysmenorrhea cause. Therefore, decreasing production of prostaglandins should be taken into account in developing its medicine. Non-steroidal drugs are considered as a typical remedy for menstrual pain which can cause side effects (9). Thymus vulgaris has antispasmodic effect and does not have gastrointestinal complication, also it relieves gastrointestinal disorders such as ulcers, indigestion, constipation, flatulence, and asthma (11). Our finding demonstrated that thymus vulgaris as well as ibuprofen significantly reduced primary dysmenorrhea compared with placebo. Iravani et al. showed that all three treatments (placebo, thymus vulgaris, 1% and 2%) reduced the severity of dysmenorrhea but the most effective treatment was thymus vulgaris 2% (15).

In another study using thymus vulgaris and ibuprofen decreased the menstrual pain with similar score at the first and the second months of trial (16). Rouzbahani et al. used thymus vulgaris and mefenamic acid on primary dysmenorrhea that showed the mean pain intensity before treatment was not significantly different between the groups. After medication, the mean pain intensity was reduced significantly in both groups, however, no significant difference was observed in reducing pain measure between the thymus vulgaris and mefenamic acid groups (17). Jaffari et al. found the analgesic effect of antinociceptive effects of hydroalcoholic extract and essential oil of thymus vulgaris (18).

Some mentioned studies confirm the analgesic and antispasmodic effects of thymus vulgaris which have been indicated in the traditional medicine. In our study, thymus vulgaris also was able to significantly reduce pain and spasm in our patients involved with dysmenorrhea remarkably, hence, our finding is consistent with the findings of the above studies. It is worth mentioning that a similar study has not been conducted on the effects of thymus vulgaris on systemic symptoms and menstruation volume in Iran and abroad, however, some studies have been performed about other herbal medicine.

In our study, bleeding in each of the three groups showed no significant difference before and following treatment. Other study did not demonstrate differences in the intensity of bleeding in valerian (valeriana officinalis) group versus placebo (19). In Modarres et al.’s study, mefenamic acid has been more effective in reducing menstrual bleeding compared to chamomile (20). In the present study, the systemic signs intensity showed no significant differences in any of the three treatment groups before and after treatment except in variables ;nausea, vomiting, diarrhea, and fatigue. Mirabi et al. showed that the mean total score of the systemic symptoms associated with dysmenorrhea decreased compared to pre-treatment, however, this reduction was not statistically significant between valerian and placebo, except for the intensity of faint (19). Torkzahrani et al.’s study showed that the total score of the systemic symptoms associated with dysmenorrhea declined compared to pre-treatment, however, this reduction was not statistically significant between the study and control groups except for the intensity of lethargy in which the difference was significant (21). We investigated two different antispasmodic medicines (herbal and synthetic) and compared to placebo, while the above queries compared an antispasm medicine with placebo. We comprehend that maybe this is the essential difference of our study with them. Our study indicated that majority of the subjects in thymus vulgaris group found the treatment excellent, while the ibuprofen group considered a moderate effect.

Omidvar et al. studied the effect of fennel on pain intensity in dysmenorrhoea and found 52 percent of patients in the study group (vs. 8% placebo) considered the effect of treatment excellent (22). Iravani’s study revealed that the effect of thymus vulgaris 2% in the reduction of the severity of dysmenorrhea was more than thymus vulgaris 1% (15). The effect of thymus vulgaris 2% was more than ibuprofen in our study, however, other research may have done the best dose of thymus vulgaris on dysmenorrhea, bleeding reducing and systemic symptoms. The weakness of our study is that we used visual analogue scale to evaluate pain intensity and each person's perception of pain is different, thus, our study subjects may have rated the same pain score differently. On the other hand, as VAS was the only scale available, we had no choice but to use it.

In conclusion, herbal medicines have fewer side effects versus synthetic drugs. Nonsteroidal anti-inflammatory drugs are effective in the treatment of primary dysmenorrhea, however, not complication-free. So, the researchers are increasingly using complementary and alternative medicine approaches. Based on our study, the essence oil of thymus vulgaris 2% relieved our patients’ primary dysmenorrhea notably, as well as ibuprofen, owing to anti-prostaglandin and antispasmodic of thymus vulgaris was also seen in the ibuprofen group. It is suggested that clinical studies of this herbal drug should be done comparing with other NSAIDs drugs to reduce dysmenorrhea.

## Notes

**Funding: **This study was supported by a research grant from the Babol University of Medical Sciences (Grant Number: 9031111).
